# The Combined Effect of Environmental Policies on China’s Renewable Energy Development: A Multi-Perspective Study Based on Semiparametric Regression Model

**DOI:** 10.3390/ijerph20010184

**Published:** 2022-12-23

**Authors:** Xiaolei Yang, Shuiying Zhong

**Affiliations:** Economics and Management School, Wuhan University, Wuhan 430072, China

**Keywords:** green supervision and public regulations, green standardized regulations, renewable energy development, semiparametric regression model

## Abstract

Based on a sample of 92 listed renewable energy enterprises in China from 2007–2017, this paper empirically examines the nonlinear effect of environmental policies on renewable energy investments using a semiparametric regression model. Environmental policies are divided into three groups in terms of pre-control, in-process governance, and post-accounting—the groups being green supervision and public regulations, green standardized regulations, and green accounting regulations—and this paper explores the differences in the effects of environmental policies at different stages. The results indicate that the relationship between environmental policies and renewable energy development has been unstable, following a “W-shaped” pattern. Green supervision and public regulations can greatly enhance investments in the renewable energy industry, with an estimated coefficient of 10.8173. Green standardized regulations have a similar “W-shaped” impact on renewable energy development. However, the nonlinear impact of green accounting regulations on renewable energy development fails the significance test. In addition, the effect of environmental policies on investment in the solar energy industry is positive, with a coefficient of 1.0697. The positive effect of environmental policies on investments in the renewable energy industry is reflected mainly in medium-, small-, and micro-sized enterprises. These findings contribute to the literature on the effectiveness of environmental policies by putting a set of environmental policies into a unified framework to explore their combined effects.

## 1. Introduction

A country’s development is inseparable from energy consumption. However, the sustainable development of a country can be restricted due to limited energy sources [1,2]. Therefore, an energy transition is imperative under the circumstances. The International Energy Agency (IEA) states that energy transition is a driving force for CO2 emissions reduction, which can be achieved by promoting the development of renewable energy, hydrogen, and sustainable biomass. Specially, it was reported in Energy Outlook 2022 by the IEA that in order to achieve the goal of net zero emissions by 2050, the shares of renewable energy generation and renewable energy consumption must increase from 26% and 19% in 2019 to 90% and 79% in 2050. Furthermore, the International Renewable Energy Agency (IRENA) further points out that hydrogen should account for 12% of global energy use by 2050.

In practice, China is a country with a large energy demand, but insufficient stock. Energy constraints can hinder economic and social development. Therefore, the use of renewable energy resources is a necessity. A few scholars have found that the lack of funds is the biggest obstacle to the development of the renewable energy industry [3,4,5,6], which highlights the importance of investments in the renewable energy industry. A large number of environmental policies in China have been implemented accordingly, including green financial incentives, sewage charges, etc. Existing studies have focused on the effect of single policies [7,8]; however, in practice, these policies jointly affect the operation of the whole economic system and may work synergistically or against each other. Therefore, the combined effects of these policies deserve our attention. However, there is limited causal evidence of this important issue. Our paper approaches this question by putting the environmental policies aimed at promoting green development under a unified framework to explore their combined effects, and comparing the effects of the policies at different stages in order to provide a policy reference for the government to promote the development of a green economy.

Based on a sample of 92 listed renewable energy enterprises in China from 2007–2017, this paper empirically examines the nonlinear effect of environmental policies on renewable energy investments using a semiparametric regression model. This paper divides a set of environmental policies into three groups in terms of pre-control, in-process governance, and post-accounting—the groups being green supervision and public regulations, green standardized regulations, and green accounting regulations—and further explores the differences in the effects of environmental policies at different stages. The results indicate that the relationship between environmental policies and renewable energy investments may be a “W-shaped” relationship, which is mainly manifested in green standardized regulations. In addition, green supervision and public regulations can greatly enhance investments in the renewable energy industry, but green accounting regulations cannot. Moreover, the effects of environmental policies differ with different industry types and business scales.

This paper contributes to research topics. First, this study is related to a large body of literature analyzing the effectiveness of environmental policies. Most scholars, in fact, have focused on the effects of a single environmental policy [9,10,11], but have neglected the combined effects of these policies. For example, Nie et al. (2016) observed that green financial incentives can enhance environmental awareness, and thus increase the demand for and output of renewable energy [12]. Internationally, analyses of the impacts of single environmental policies in different countries on the development of the renewable energy industry have been conducted [13,14,15,16]. This paper thus distinguishes itself from previous work by putting environmental policies aimed at promoting green development under a unified framework to explore their combined effects. Furthermore, these policies are divided into different groups in terms of pre-control, in-process governance, and post-accounting, and the differences in the effects of environmental policies at different stages are analyzed.

Second, the previous literature has documented the responses of heavily polluting enterprises to environmental regulation [17,18,19]. For example, Zhao and Xiong (2010) asserted that economic incentives can generate a positive effect on improving the development of industrial enterprises [20]. Zhang et al. (2011) and Sun and Yang (2017) insisted that environmental taxes will enhance economic efficiency in motivating polluters to actively reduce pollution emissions [21,22]. However, there is a lack of attention on renewable energy enterprises. As environmentally friendly enterprises, their exposure to environmental regulation also deserves our attention. Different from previous work, this paper considers renewable energy enterprises as the research object to investigate their responses to environmental policies. This paper also improves our understanding of different response patterns of enterprises in different industries to environmental policies.

Third, although a large number of studies have confirmed the effectiveness of environmental policy governance, the responses of micro-subjects when facing policy interventions are always flexible rather than following a simple linear form. The previous literature has been based on linear and grouped research methods [23], which do not consider the possible complex relationships in the effects of policies. For example, the fixed-effect and DID models have been adopted to test the impacts of environmental policy [24,25,26]. To address this gap, this study uses a semiparametric regression model to explore the possible nonlinear effects of environmental policies.

The remainder of the paper is organized as follows: Section 2 presents the paper’s theoretical basis and hypothesis. Section 3 details the models and variable selection. Section 4 analyzes the combined impacts of environmental policies on renewable energy development in different stages. Section 5 explores the different effects from a classification perspective. Section 6 concludes the paper.

## 2. Mechanism of Environmental Policies’ Effects on Renewable Energy Development

Environmental policies are aimed at promoting regional green development, which is reflected in an increase in the share of renewable energy in primary energy consumption. Therefore, exploring the impact of environmental policies on renewable energy development can help to achieve sustainable development; however, the effects differ at different stages of environmental policies. We therefore divide environmental policies into the categories of green supervision and public regulations, green standardized regulations, and green accounting regulations, in terms of pre-control, in-process governance, and post-accounting.

(1) Green supervision and public regulations refer to the supervision of enterprises’ access to environmental policy preferences. This category is usually strict in monitoring standards. Enterprises that do not fit within the scope of the policy are excluded, guaranteeing that resources are precisely utilized in the environmentally friendly renewable energy sector, rather than in other sectors. This avoids the waste of resources and improves the quality and efficiency of renewable energy development. Therefore, we propose Hypothesis 1.

**Hypothesis** **1.** *Green supervision and public regulations can promote renewable energy development.*

(2) Green standardized regulations refer to a set of policy benefits provided by the government to environmentally friendly renewable energy enterprises, such as green finance, etc. These policies are of great significance for providing funding for the renewable energy industry to address its financing difficulties. Furthermore, they include some penalties for highly polluting industries, such as environmental taxes. These will enhance enterprises’ attention to renewable energy and guide capital into the renewable energy industry. Therefore, we propose Hypothesis 2.

**Hypothesis** **2.** *Green standardized regulations can promote renewable energy development.*

(3) Green accounting regulations integrate loss of resources and environmental damage into national economic statistics, and provide accurate green economic information for a country. This allows the country to gain real green economic growth not at the expense of the environment, in order to further develop plans for better promoting green development. Since green accounting regulations are only a post-accounting behavior adopted by the government, they have a limited effect on promoting renewable energy development. Therefore, we propose Hypothesis 3.

**Hypothesis** **3.** *Green accounting regulations produce a limited effect on renewable energy development.*

The roles of total environmental policies in renewable energy development can be complex from a comprehensive view. Hypothesis 4 is thus proposed.

**Hypothesis** **4.** *Total environmental policies produce a complex effect on renewable energy development.*

**Figure 1:**
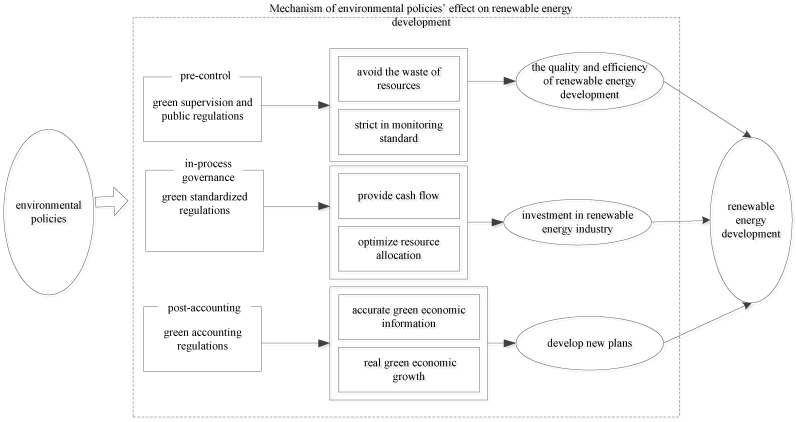
Mechanisms of environmental policies’ effects on renewable energy development.

Figure 1 sums up the mechanisms of environmental policies affecting renewable energy development at different stages.

## 3. Data and models

### 3.1. Sample Selection

This paper selects renewable energy enterprises in China’s A-share market from 2007 to 2017 as samples. Enterprises related to the exploitation and utilization of renewable energy are considered as renewable energy enterprises in this paper. Some enterprises were further removed according to the following principles: Firstly, enterprises that were listed in China’s A-share market after 31 December 2010 were removed; secondly, ST, *ST, and PT enterprises were excluded. In addition, enterprises with incomplete data were dropped. In total, 92 enterprises were selected accordingly, composed of 42 solar energy enterprises, 22 wind energy enterprises, 11 biomass energy enterprises, 7 geothermal energy enterprises, 6 hydro energy enterprises, and 4 other enterprises connected with the development of renewable energy.

### 3.2. Variables

Many factors related to the enterprise itself will affect investments in the renewable energy industry. Wang and Song (2015) asserted that the resource endowment of an enterprise affects the enterprise’s investment decisions [27]. Palangkaraya et al. (2009) and Zhou (2010) indicated that the economic efficiency of an enterprise can be affected by enterprise age [28,29]. In addition, the value of Tobin’s Q can be an indicator of the development potential of an enterprise, and can also be used to capture the investment opportunities of enterprises [30,31]. Specifically, the higher the value of Tobin’s Q, the stronger the investment demand of an enterprise. The control variables include the resource endowment of enterprises, enterprise age, and development potential. The relevant variables are described in Table 1.

(1) Renewable energy development INV. Man et al. (2013) found that this variable can be defined as the investments of renewable energy enterprises. Two definitions therefore emerge [32]. Firstly, external investment refers to the direct purchase of securities issued by external business entities, such as treasury bills, special treasury bonds, local bonds, and enterprise bonds, all of which have nothing to do with the renewable energy industry. Internal investment is the money used to add assets in renewable energy enterprises. Since the main business of renewable energy enterprises is related to new energy and renewable energy, internal investment is able to capture investment in renewable energy to the greatest extent. This paper therefore measures renewable energy development by the capital used for adding permanent assets, intangible assets, and other long-term assets in renewable energy enterprises, which is consistent with the research of Li and Yang (2015) [30].
(2) Environmental policies ENV. More scholars focus on a single policy and its environmental and economic effects. Therefore, there is a lack of attention on the comprehensive effects of a set of environmental policies. Drawing on the work of Yang et al. (2021), this research therefore provides a new indicator, the “green institutional environment”, for quantitative analysis using the functional data analysis method and the functional entropy weight method. This is because there is a set of complex indicators in green institutional environmental systems, and the importance of each indicator will vary over time due to complicated and dynamic economic effects.

As with Yang et al. (2021), we find that environmental policies can be further divided into three groups (i.e., GSPI, GSI, and GAI) in terms of pre-control, in-process governance, and post-accounting [33]. These three groups of environmental policies, GSPI, GSI, and GAI, are, respectively, represented by three indices (i.e., environmental labeling institutions, green market access institutions, and environmental information disclosure institutions), five indices (i.e., performance evaluation institutions, environmental management institutions, green economic policy, green technology innovation institutions, interregional environmental protection coordination, and benefit balance institutions), and four indices (i.e., green audit, green accounting, green GDP, and emission trading market), which can be obtained using Model (1).

(1) $$ENV(t|t^{*})=\sum_{j=1}^{m}w_{j}(t|t^{*})\cdot x_{j}(t),\;t_{0}\leq t\leq t^{*}$$

where $x_{j}(t)$ is policy indicators. $w_{j}(t|t^{*})$ is the dynamic weight curve of policy indicators at each time node. With the passage of time nodes, $w_{j}^{t^{*}}$ is updated constantly, thus forming a new indicator for environmental policy closely related to $t^{*}$. Accordingly, the maximum and minimum values of these three kinds of environmental policies are 0.000 and 0.025, 0.2 and 0.8, and 0.000 and 0.030, respectively, which represent the degree of development of environmental policies in terms of pre-control, in-process governance, and post-accounting. The environmental policies from the perspective of in-process governance are well developed. For more information about relevant policy indicators $x_{j}(t)$, such as their sub-indicators and calculation process, refer to Yang et al. (2021) [33].

(3) Control variables. The resource endowment of an enterprise is measured by its asset:liability ratio. Time from the listing year to the sample period is used as a measure of enterprise age. Development potential is represented by the value of Tobin’s Q.

The sources of business data are the CCER database and CSMAR database. The data for the green institutional environmental index are derived from the Enterprise Environmental Information Disclosure Index (2014–2017), Annual Statistic Report on Environment in China (2007–2017), China Statistical Yearbook on Science and Technology (2007–2017), National Bulletins on Water Resources (2007–2017), China Health Statistical Yearbook (2007–2017), China Statistical Yearbook (2007–2017), the Clean Development Mechanism in China, and the 2007–2017 Annual Reports and Social Responsibility Reports of Banks.

### 3.3. Descriptive Statistics

The descriptive statistics of each variable within the sample interval are shown in Table 2.

In Table 2, within the sample interval, it can be seen from the maximum value, minimum value, and standard deviation of renewable energy development that the data for renewable energy development are scattered. Furthermore, the skewness and kurtosis of renewable energy development are 6.7107 and 54.8628, respectively, which indicate that the sample data present a right-skewed distribution with a sharp peak.

Second, the average value of environmental policy indicators is close to its median value. It can also be seen from the small standard deviation that there is little difference in the sample data. The skewness and kurtosis of environmental policy indicators are −0.1993 and −1.2458, respectively, which indicate that the sample data present a left-skewed distribution with a flat shape.

Third, in terms of resource endowment and enterprise age, the average value is relatively close to the median value and the absolute numbers of skewness and kurtosis are small, displaying a symmetrical distribution of data. In terms of development potential, the skewness is 3.1969, which indicates that the sample data present a right-skewed distribution. It can also be seen from the small standard deviation that there is little difference in the development potential of most sample enterprises.

### 3.4. Models

The environmental policies studied in this paper include a set of policies aimed at promoting green development. Different policies may impose different effects on renewable energy development. The impact of the environmental policy system on renewable energy development is therefore complex and nonlinear. As an approach between parametric and nonparametric models, the semiparametric regression model has the following advantages: it not only eliminates problems such as improper model settings that may exist in parametric models, but also overcomes the problem of dimensionality brought by nonparametric models (Wu et al., 2018) [34]. The semiparametric method has therefore been adopted to investigate the nonlinear effect of the environmental policy system, which is consistent with the research of Zheng and Ye (2015) [35].

The semiparametric method is adopted to measure the complex nonlinear relationship between the environmental policy system and renewable energy development. The natural logarithm of INV and AGE has been taken. The model is shown in (2).

(2) $$LNINV_{it}=\alpha_{0}+G(ENV_{it})+\alpha_{1}RE_{it}+\alpha_{2}LNAGE_{it}+\alpha_{3}TQ_{it}+\varepsilon_{it}$$

where enterprise is represented by i and the year is expressed by t. $INV_{it}$ represents renewable energy development, $ENV_{it}$ represents environmental policies, and the nonlinear effect studied in this paper is expressed by G(·). $RE_{it}$, $AGE_{it}$, and $TQ_{it}$ are the enterprise’s resource endowment, the age of the enterprise, and development potential. $\alpha_{1}$, $\alpha_{2}$, and $\alpha_{3}$ are the coefficients of the effect of the enterprise’s resource endowment, age, and development potential on investment in renewable energy. $\alpha_{0}$ is intercept terms; $\varepsilon_{it}$ is random disturbance variables.

In fact, the semiparametric method can not only be applied to the exploration of nonlinear relationships, but can also help to test linear relationships. Therefore, from the perspective of environmental policies at different stages, the models can be constructed as follows.

(3) $$LNINV_{it}=\beta_{0}+G(GSPI_{it})+\beta_{1}RE_{it}+\beta_{2}LNAGE_{it}+\beta_{3}TQ_{it}+\upsilon_{it}$$

(4) $$LNINV_{it}=\chi_{0}+G(GSI_{it})+\chi_{1}RE_{it}+\chi_{2}LNAGE_{it}+\chi_{3}TQ_{it}+\mu_{it}$$

(5) $$LNINV_{it}=\delta_{0}+G(GAI_{it})+\delta_{1}RE_{it}+\delta_{2}LNAGE_{it}+\delta_{3}TQ_{it}+\omega_{it}$$

where $GSPI_{it}$ represents green supervision and public regulations, $GSI_{it}$ represents green standardized regulations, and $GAI_{it}$ represents green accounting regulations.

## 4. Empirical Estimates

### 4.1. Estimation of Long-Term Relationships between Variables

The results of the unit root test are shown in Table 3, indicating that all the variables are stationary at the 5% significance level.

Next, the results of a cointegration test are presented in Table 4, showing equilibrium between the related variables.

### 4.2. Estimates of the Nonlinear Impact of Environmental Policies on Renewable Energy Development

**Figure 2:**
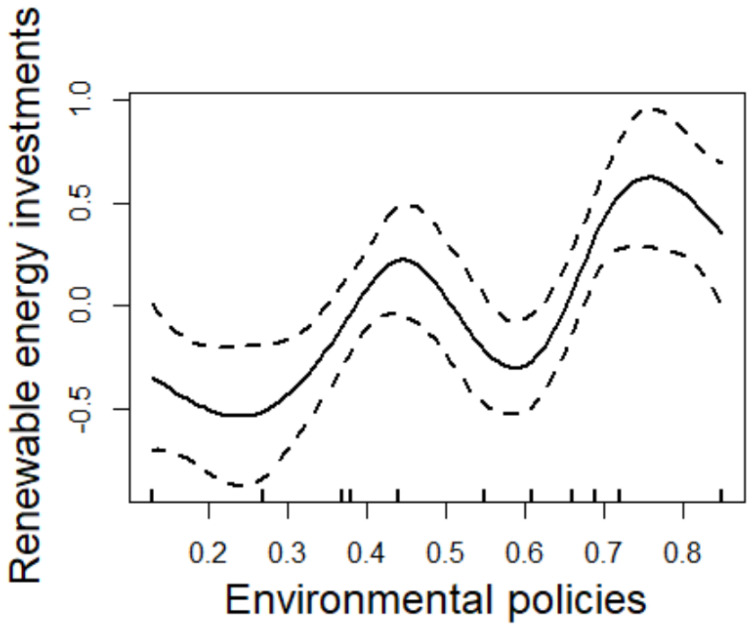
Nonlinear effect of environmental policies on investment in renewable energy enterprises.

The results of the nonlinear impact are shown in Table 5 and Figure 2.

This paper draws several conclusions based on the tables and figures above:

(1) There is a “W-shaped” relationship between the environmental policy system and renewable energy development at the 10% significance level, which verifies the rationality of the nonparametric terms of the model and further confirms the superiority of the semiparametric regression model. This result is also consistent with Hypothesis 4.

This phenomenon may be due to the double effect of environmental policies on renewable energy development. On the one hand, various environmental policies promote the green transformation of enterprises by enriching financing channels, paying environmental taxes, raising pollution discharge standards, and strengthening the responsibility of polluters. To be specific, taking financing channels as an example, green subsidies can drive enterprises to reduce high-polluting activities through direct funding, while green credit provides special loans for energy conservation and environmental-protection projects. These policies undoubtedly promote investment in renewable energy. On the other hand, the diversity and complexity of environmental policies may lead to a problem, i.e., the excessive implementation of policies. However, due to financing difficulties, high investment risk, and long return periods, investment in renewable energy enterprises is highly sensitive to the impacts of environmental policies. Therefore, excessive implementation of environmental policies inhibits renewable energy development. The impact of the environmental policy system on renewable energy development depends on the magnitude of these two effects. When the first effect is greater than the second effect, environmental policies will promote investment in renewable energy. When the second effect is greater, environmental policies will inhibit renewable energy development. Furthermore, this paper finds that environmental policies have an unstable effect on investment in renewable energy enterprises. This is because there are a large number of policies that have not been implemented in the environmental-policy system, which is extremely unstable. In addition, the policies may interact with each other, which means that the effects may be amplified or weakened.

(2) The control variables discussed above show that the resource endowment of enterprises is an inverse indicator; it significantly inhibits investment in renewable energy. This is because an enterprise’s resource endowment is limited, and the enterprise needs to increase production size and market share through debt management. Most well-developed enterprises have the ability to use debt from creditors to implement high-efficiency production. Therefore, the debt ratio of an enterprise represents its vitality. Enterprises with high debt ratios (i.e., insufficient resource endowments) tend to be more competitive and more reliant on high-risk, high-yield investments, such as renewable energy development. This indicates that China’s renewable energy enterprises should appropriately increase their debt ratios in exchange for greater profit margins and stronger business vitality. Second, the coefficient of enterprise age on renewable energy development fails the significance test. This is because the renewable energy industry, as an emerging industry, is still in its infancy. Enterprise leaders pay more to operations than investments. Therefore, enterprise age does not generate a significant effect on renewable energy development. Third, development potential inhibits the level of renewable energy development at the 1% significance level. This could be because renewable energy enterprises with higher development potential spend more money on technology promotion instead of investment, resulting in a lower level of renewable energy development.

### 4.3. Estimates of the Impact of Environmental Policies on Renewable Energy Development at Different Stages

Environment policies were divided into three groups, i.e., green supervision and public regulations, green standardized regulations, and green accounting regulations, in terms of pre-control, in-process governance, and post-accounting. This paper further explores the impact of these three kinds of policies on renewable energy development.

We can see from the results that green standardized regulations can generate a significant nonlinear effect on investment in renewable energy enterprises. The nonlinear relationship between green accounting regulations and investments in renewable energy enterprises fails the significance test. A significant linear effect exists between green supervision and public regulations and investments in renewable energy enterprises. Based on this effect, this paper further uses a panel data model to test the linear relationship between green supervision and public regulations and renewable energy development, as shown in (5). A Hausman test was therefore conducted, whose results indicate that a fixed-effect model should be constructed (chi-sq value is 19.26; *p*-value is 0.0017).

(6) $$LNINV_{it}=C_{0}+C_{1}ENV_{it}+C_{2}RE_{it}+C_{3}LNAGE_{it}+C_{4}TQ_{it}+\varsigma_{it}$$

**Figure 3:**
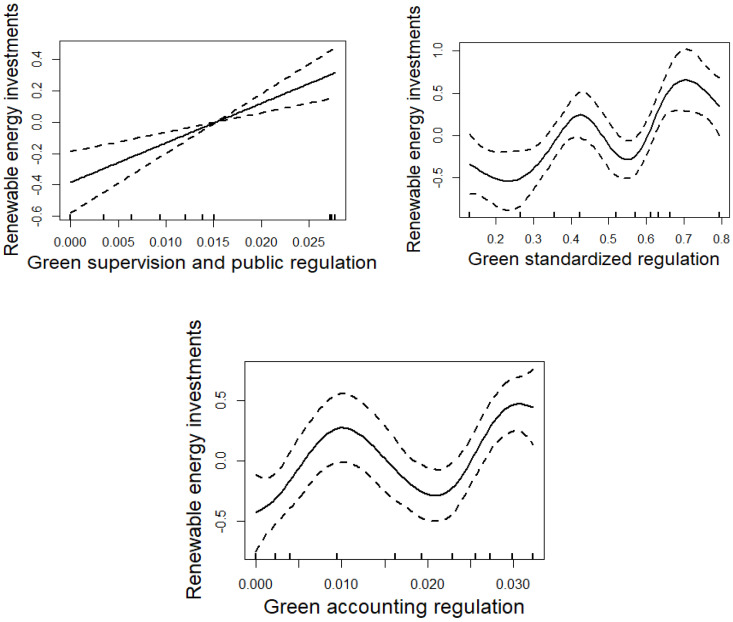
Effect of three kinds of environmental policies on investments in renewable energy enterprises.

The results are shown in Table 6 and Figure 3.

This paper draws several conclusions from the table and figure above:

(1) Green supervision and public regulations can enhance investments in the renewable energy industry, with an estimated coefficient of 10.8173. The nonlinear relationship between green accounting regulations and investments in the renewable energy industry fails the significance test. This shows that green supervision and public regulations can play an important role in enhancing investment in the renewable energy industry, but green accounting regulations cannot, which is consistent with Hypothesis 1 and 3.

This may be because green supervision and public regulations eliminate pollution from the source; however, green accounting regulations work mainly from the perspective of post-accounting, incorporating environmental information into the accounting system to provide the country with accurate green economic information. The importance of eliminating pollution beforehand has been recently emphasized by the government, and is thus playing a strong role in promoting investment in renewable energy. However, while green accounting regulations have a promising future and short implementation time, unclear implementation methods and immature system construction will lead to difficulties in promoting renewable energy development.

(2) A nonlinear effect of green standardized regulations on investment in the renewable energy industry can be found, with a curve shape similar to the “W-shape”. It indicates that the nonlinear effect of environmental policies on renewable energy development is reflected mainly in green standardized regulations, which is inconsistent with Hypothesis 2. Due to serious pollution and irreversible resource depletion, China attaches great importance to the governance of environmental problems. Green standardized regulations include rules to regulate and restrain the economic activities of various actors, which are therefore expected to be the main component of environmental policies. The number of policies in green standardized regulations are much more than the number in the other two kinds of environmental policies. Different policies may lead to different effects on renewable energy development. Some policies in green standardized regulations were issued earlier and have been implemented for longer, resulting in the significant promotion of renewable energy development. However, some policies produce limited promotion effects. This results in an unstable relationship between green standardized regulations and renewable energy development.

The results are summarized in Table 7.

## 5. Estimation from the Classification Perspective

### 5.1. Group Estimation Based on Industry-Type Classification

The impact of environmental policies on investments in renewable energy enterprises differs with different business scales and industry types. The conclusions of this paper are of great significance for objectively focusing policies and coordinating them to promote the development of renewable energy enterprises.

In fact, different renewable energy types are valued differently by the government. Therefore, there are significant differences in the development status of the different energy types. Based on this fact, this paper tries to explore the relationship between environmental policies and renewable energy development with different energy types. The sample enterprises include five energy types: solar energy, hydro energy, biomass energy, wind energy, and geothermal energy. Model (2) is created accordingly. A linear effect of environmental policies on investment in renewable energy enterprises with different energy types is obtained and Model (6) is thus estimated.

A Hausman test was therefore conducted, whose results indicate that fixed-effect models and random-effect models should be constructed separately (chi-sq values are 11.94, 3.7246, 7.3824, 15.8025, and 0.2422; *p*-values are 0.0357, 0.4446, 0.1170, 0.0033, and 0.9932). The results are shown in Table 8.

From Table 8, this paper draws several conclusions:

(1) Environmental policy will promote investments in solar energy with a coefficient of 1.0697, at the 10% significance level. This is because China has rich solar energy resources and huge development potential. In addition, the development of China’s solar energy industry is occurring relatively late. It is thus highly dependent on policies and is highly valued by the government. Specifically, the feed-in tariff on photovoltaic power issued in 2011 can help to improve China’s solar power market. The 13th Five-Year Plan for Solar Energy Development in 2016 can help diversify solar power generation methods and build a clean and efficient energy system. These all reflect the government’s strong support for the development of the solar energy industry, which has achieved a desirable effect.
(2) Environmental policy will inhibit investments in biomass energy with a coefficient of −1.2101, at the 10% significance level. This indicates that China’s environmental policy is still unable to effectively promote biomass, and may even generate a negative impact, though the importance of biomass energy in the energy supply of China is increasing. This may be because, on the one hand, there are still some problems in the development of biomass energy itself, such as an inconsistent understanding of biomass energy, less development experience, and inadequate technical expertise. On the other hand, China’s policy system has not yet been perfected regarding the biomass energy industry, which is manifested in scattered policies. In addition, policies are mostly aimed at other industries instead of the biomass energy industry, which sends a signal to the public, resulting in the inflow of social capital into other industries instead of the biomass energy industry.
(3) The coefficients of environmental policies on investments in wind energy, hydro energy, and geothermal energy fail the significance test. This is due to limited developmental conditions. Due to natural conditions such as geographical location and climate change, the development of China’s hydropower industry has stagnated. As far as the wind energy industry is concerned, the utilization of wind energy in China is still stagnant, and even faces the emergence of wind curtailment problems in many regions. In terms of the geothermal energy industry, due to its requirements for large capital investment and strong technical expertise, its development is not likely, but its potential is still very large.

### 5.2. Group Estimation Based on Business Scale Classification

According to the “Statistical Measures for the Classification of Enterprises as large-, medium-, small- and micro-scale”, 92 enterprises were further divided into two categories by enterprise scales. Model (2) is estimated separately. The results show that environmental policies can generate a linear effect on investment in renewable energy enterprises regarding different enterprise scales. Based on this information, Model (6) is further estimated. A Hausman test was therefore conducted, whose results indicate that fixed-effect models and random-effect models should be constructed separately (chi-sq values are 23.0180 and 3.1290; *p*-values are 0.0001 and 0.5365). The results are shown in Table 9.

From Table 9, this paper finds that environmental policies will promote investments in medium-, small-, and micro-sized enterprises with a coefficient of 1.8276. However, the coefficient of the effect of environmental policies on investments in large enterprises fails the significance test. This is because an enterprise’s scales can affect its ability to raise capital and its business strategy. Specifically, due to economies of scale, larger enterprises have greater flexibility in their business strategies, a greater ability to take risks, lower financing costs, and less dependence on policies. The problems facing medium-, small-, and micro-sized enterprises, such as financing difficulty, can lead to poor external financing ability and greater dependence on policies. Moreover, medium-, small-, and micro-sized enterprises tend to be more competitive in order to increase production size and market share, which makes their business strategies more radical than that of large enterprises and more dependent on “high-risk, high-yield” investment such as renewable energy to consolidate their market base and gain benefits. Therefore, the effect of environmental policies on investment in medium-, small-, and micro-sized enterprises is greater.

## 6. Conclusions and Discussions

This paper empirically examines the combined effect of environmental policies on renewable energy investments based on a sample of 92 listed renewable energy enterprises in China from 2007–2017. Using a semiparametric regression model, this paper shows that the combined effect of environmental policies on renewable energy development has been unstable, showing a “W-shaped” pattern. Furthermore, this paper divides a set of environmental policies into three groups in terms of pre-control, in-process governance, and post-accounting—those groups being green supervision and public regulations, green standardized regulations, and green accounting regulations—and further finds significant differences in the effects of environmental policies at different stages. Specifically, the “W-shaped” relationship is mainly manifested in green standardized regulations. Green supervision and public regulations can produce a promotional effect, but green accounting regulations cannot. Moreover, significant differences in the effects of environmental policies with different industry types and business scales can be observed. Specifically, the positive effect of environmental policies on investments in the renewable energy industry is reflected mainly in the solar energy industry and medium-, small-, and micro-sized enterprises.

Drawing from the empirical results, this paper sheds fresh light on the development process of environmental policies at different stages. In terms of green supervision and public regulations, the importance of eliminating pollution beforehand has been recently emphasized by the government, and thus plays a strong role in promoting investment in renewable energy. Green standardized regulations, as the main component of environmental policies, include a great number of rules and regulations. However, these policies may work against each other, which results in an unstable effect on renewable energy development. The government thus needs to prioritize the rationality of policies, i.e., by avoiding excessive implementation of policies and maintaining the stability and continuity of policies. In contrast, green accounting regulations have a promising future, but few policies have been implemented thus far, meaning that they do not play a significant role in promoting renewable energy development. This situation deserves more attention from local governments, including investing more into indicator design and system construction.

In addition, environmental policies produce effects on all aspects of social life, not just renewable energy development. Impacts on economic development and environmental quality can be further explored in follow-up research. Furthermore, this research has a certain degree of universal applicability. A new research framework of dividing environmental policies into different groups was adopted in this paper to explore the different effects of policies. As a result, the presented research framework and ideas can not only provide a theoretical reference and academic reference for future research, but can also be applied to the analysis of environmental policies in other countries.

## Figures and Tables

**Table 1:** Description of main variables and indicators.

Variable	Symbol Indicator Description	Unit
Renewable energy development	The capital used for adding permanent assets, intangible assets, and other long-term assets in renewable energy enterprises	RMB
Environmental policies	Green institutional environmental index	None
Enterprise’s resource endowment	Asset–liability ratio of renewable energy enterprises	None
Enterprise age	A period from the listing year to the sample interval	Year
Development potential	Renewable energy enterprises’ Tobin’s Q	None

**Table 2:** Descriptive statistics for each variable.

Variable	Average Value	Median Value	Minimum Value	Maximum Value	Standard Deviation	Skewness	Kurtosis
Renewable energy development	951,900,000	232,300,000	−352,200,000	27,960,000,000	2,449,148,000	6.7107	54.8628
Environmental policy indicators	0.5169	0.5540	0.1305	0.8547	0.2171	−0.1993	−1.2458
Enterprise’s resource endowment	0.5571	0.5712	0.0386	1.2008	0.1788	−0.3268	0.0490
Enterprise age	13.47	14.00	0.00	28.00	5.4130	−0.0199	−0.4987
Development potential	1.7340	1.4590	0.7978	10.1589	0.9337	3.1969	16.2706

**Table 3:** Results of unit root test.

Methods	*INV*	*ENV*	*RE*	*AGE*	*TQ*
Levin, Lin, and Chu t *	−18.0261 ***	−32.5115 ***	−12.6475 ***	−53.7304 ***	−28.8035 ***
	(0.0000)	(0.0000)	(0.0000)	(0.0000)	(0.0000)
Im, Pesaran, and Shin W-stat	−2.23335 **	−14.6464 ***	−1.69587 **	−143.718 ***	−6.35045 ***
	(0.0128)	(0.0000)	(0.0450)	(0.0000)	(0.0000)
ADF—Fisher Chi-square	259.720 ***	500.201 ***	237.827 ***	1694.70 ***	404.563 ***
	(0.0001)	(0.0000)	(0.0046)	(0.0000)	(0.0000)
PP—Fisher Chi-square	354.644 ***	1389.70 ***	269.286 ***	1694.70 ***	535.011 ***
	(0.0000)	(0.0000)	(0.0000)	(0.0000)	(0.0000)

Values in parentheses are the *p* values corresponding to each statistic. ***, ** and * indicate rejection of the null hypothesis at the 1%, 5% and 10% significance levels, respectively.

**Table 4:** A cointegration test of the related variables.

Methods	Renewable Energy Development and Environmental Policies	Renewable Energy Development, Environmental Policies, and Control Variables
Panel PP	−8.0826 ***	−3.1275 ***
	(0.0000)	(0.0009)
Panel ADF	−11.7690 ***	−2.9774 ***
	(0.0000)	(0.0015)
Group PP	−8.8801 ***	−10.2553 ***
	(0.0000)	(0.0000)
Group ADF	−12.3452 ***	−5.2328 ***
	(0.0000)	(0.0000)

*p*-values in brackets. *** *p* < 0.01.

**Table 5:** Impact of environmental policies on renewable energy development.

Variables	Coefficient	Standard Error	T Statistic	*p*-Value
RE	1.08 ***	0.32	3.35	0.00
AGE	−0.02	0.12	−0.20	0.84
TQ	−0.71 ***	0.07	−10.91	0.00
G(ENV)	/ *	0.42	−1.74	0.08

*** *p* < 0.01, * *p* < 0.1.

**Table 6:** Estimation for three kinds of environmental policies.

Variables	Semiparametric Regression Model		Variables	Fixed-Effect Model
	Green Standardized Regulations	Green Accounting Regulations		Green Supervision and Public Regulations
RE	1.07 *** (3.33)	1.11 *** (3.44)	RE	2.0992 *** (5.32)
AGE	−0.02 (−0.16)	−0.01 (−0.10)	AGE	0.4114 ** (2.39)
TQ	−0.72 *** (−10.90)	−0.69 *** (−10.73)	TQ	−0.1907 *** (−3.39)
G(ENV)	/ * (−1.86)	/ (0.21)	INDEX	10.8173 * (1.80)

Values in parentheses are the T statistics. *** *p* < 0.01, ** *p* < 0.05, * *p* < 0.1.

**Table 7:** A summary of empirical results.

Hypothesis	Whether the Hypothesis Is Confirmed or Not	Results
**Hypothesis** **1.** *Green supervision and public regulations can promote renewable energy development.*	Yes	Green supervision and public regulations can enhance investments in the renewable energy industry, with an estimated coefficient of 10.8173.
**Hypothesis** **2.** *Green standardized regulations can promote renewable energy development.*	No	There is a “W-shaped” relationship between green standardized regulations and renewable energy development.
**Hypothesis** **3.** *Green accounting regulations will produce a limited effect on renewable energy development.*	Yes	Green accounting regulations cannot play an important role in enhancing investment in the renewable energy industry.
**Hypothesis** **4.** *Total environmental policies will produce a complex effect on renewable energy development.*	Yes	A complex effect of the environmental policy system on investment in the renewable energy industry can be obtained, whose curve shape is similar to “W-shaped”.

**Table 8:** Estimation for different energy types.

Variables	Model (6)				
	Solar Energy	Hydro Energy	Biomass Energy	Wind Energy	Geothermal Energy
RE	1.1295 * (1.78)	2.7127 ** (2.13)	3.7117 *** (4.82)	−0.9459 (−1.46)	2.8103 ** (2.18)
AGE	0.1202 (0.34)	0.4062 (0.91)	0.7671 * (1.96)	1.1738 *** (3.61)	0.2021 (0.29)
TQ	−0.1933 ** (−2.23)	0.3220 (0.81)	0.0111 (0.11)	−0.2892 *** (−2.60)	−0.0636 (−0.30)
ENV	1.0697 * (1.93)	0.3925 (0.48)	−1.2101 * (−1.64)	−0.4804 (−0.84)	0.5263 (0.50)

Values in parentheses are the T statistics. ***, **, and * indicate rejection of the null hypothesis at the 1%, 5%, and 10% significance levels, respectively.

**Table 9:** Estimation for different enterprise scales.

Variables	Model (6)	
	Large-Sized Enterprises	Medium-, Small-, and Micro-Sized Enterprises
RE	−0.0540 (−0.14)	0.9702 (1.30)
AGE	0.5732 *** (2.68)	0.0027 (0.0078)
TQ	−0.2413 *** (−4.34)	−0.1190 (−1.13)
ENV	0.2208 (0.63)	1.8276 *** (2.58)

Values in parentheses are the T statistics. *** indicates rejection of the null hypothesis at the 1%significance levels, respectively.

## Data Availability

Raw data of this article are available upon request to corresponding author.

## References

[1] Bhattacharya M., Paramati S.R., Ozturk I., Bhattacharya S. (2016). The Effect of Renewable Energy Consumption on Economic Growth: Evidence from top 38 countries. Appl. Energy.

[2] Bilgili F., Koçak E., Bulut Ü. (2016). The Dynamic Impact of Renewable Energy Consumption on CO_2_ Emissions: A Revisited Environmental Kuznets Curve Approach. Renew. Sustain. Energy Rev..

[3] Arbex M., Perobelli F.S. (2010). Solow meets Leontief: Economic growth and energy consumption. Energy Econ..

[4] Salim R.A., Hassan K., Shafiei S. (2014). Renewable and non-renewable energy consumption and economic activities: Further evidence from OECD countries. Energy Econ..

[5] Inglesi-Lotz R. (2016). The impact of renewable energy consumption to economic growth: A panel data application. Energy Econ..

[6] Qi S.Z., Li Y. (2018). Threshold effects of renewable energy consumption on economic growth under energy transformation. China Popul. Resour. Environ..

[7] Shen J., Pan J.Q. (2009). Fiscal Policy Analysis of Renewable Energy Development. Contemp. Econ. Res..

[8] Yang C.H., Tseng Y.H., Chen C.P. (2012). Environmental regulations, induced R & D, and productivity: Evidence from Taiwan’s manufacturing industries. Resour. Energy Econ..

[9] Bi Q., Gu L.M., Zhang J.J. (2015). Traditional culture, environmental system and corporate environmental information disclosure. Account. Res..

[10] Li X.J., He N. (2018). Research on the influence of environmental tax on enterprise green technology innovation under regional competition. China Popul. Resour. Environ..

[11] Abid N., Ikram M., Wu J., Ferasso M. (2021). Towards environmental sustainability: Exploring the nexus among ISO 14001, governance indicators and green economy in Pakistan. Sustain. Prod. Consum..

[12] Nie P.Y., Chen Y.H., Yang Y.C., Wang X.H. (2016). Subsidies in carbon finance for promoting renewable energy development. J. Clean. Prod..

[13] Terkla D. (1984). The efficiency value of effluent tax revenues. J. Environ. Econ. Manag..

[14] Carrasco J.M., Franquelo L.G., Bialasiewicz J.T., Galvan E., Guisado R.C.P., Prats M.A.M., Leon J.I., Moreno-Alfonso N. (2006). Power-Electronic Systems for the Grid Integration of Renewable Energy Sources: A Survey. IEEE Trans. Ind. Electron..

[15] Abadie L.M. (2009). Valuation of Long-Term Investments in Energy Assets under Uncertainty. Energies.

[16] Dent C.M. (2015). China’s renewable energy development: Policy, industry and business perspectives. Asia Pac. Bus. Rev..

[17] Porter M.E., Linde C.V.D. (1995). Toward a New Conception of the Environment-Competitiveness Relationship. J. Econ. Perspect..

[18] Bi Q., Yu L.C. (2016). Environmental Taxes, Media Surveillance and Corporate Green Investments. Financ. Account. Mon..

[19] Carfora A., Pansini R.V., Romano A.A., Scandurra G. (2018). Renewable energy development and green public policies complementarities: The case of developed and developing countries. Renew. Energy.

[20] Zhao Y.Q., Xiong N.J. (2010). Review on Economic Incentive Policies for Renewable Energy in China. Res. Econ. Manag..

[21] Zhang C., Lu Y., Guo L., Yu T.S. (2011). The intensity of environmental regulation and the progress of production technology. Econ. Res. J..

[22] Sun W.Y., Yang Q. (2017). Environmental regulation and optimal decision-making of enterprises: A study based on the mixed oligopoly model. Commer. Res..

[23] Liu G., Yang Z., Zhang F., Zhang N. (2022). Environmental tax reform and environmental investment: A quasi-natural experiment based on China’s Environmental Protection Tax Law. Energy Econ..

[24] Cao J., Ho M.S., Ma R., Teng F. (2021). When carbon emission trading meets a regulated industry: Evidence from the electricity sector of China. J. Public Econ..

[25] Li Z., Wang J. (2022). Spatial spillover effect of carbon emission trading on carbon emission reduction: Empirical data from pilot regions in China. Energy.

[26] Zhang C., Zhou D., Wang Q., Ding H., Zhao S. (2022). Will fiscal decentralization stimulate renewable energy development? Evidence from China. Energy Policy.

[27] Wang B., Song C.X. (2015). Resource Endowment, Resource Demand and Industrial Investor Introduction of Start-Up Enterprises-Evidence from GEM Listed Companies. Account. Res..

[28] Palangkaraya A., Stierwald A., Yong J. (2009). Is Firm Productivity Related to Size and Age? The Case of Large Australian Firms. J. Ind. Compet. Trade.

[29] Zhou W.X. (2010). Overinvestment or Underinvestment-Evidence from A-Share Listed Companies. China Ind. Econ..

[30] Li F.Y., Yang M.Z. (2015). Will Economic Policy Uncertainty Constrain Corporate Investment?-An Empirical Study Based on China’s Economic Policy Uncertainty Index. J. Financ. Res..

[31] Wang Q., Kwan M.P., Fan J., Zhou K., Wang Y.F. (2019). A study on the spatial distribution of the renewable energy industries in China and their driving factors. Renew. Energy.

[32] Man X.Y., Zhu X.J., Chen J. (2013). A Study on the Statistical Index System of Energy Investment. Stat. Res..

[33] Yang X., He L., Tian S., Wang D. (2021). Construction of China’s Green Institutional Environmental Index: Using Functional Data Analysis method. Soc. Indic. Res..

[34] Wu X.P., Gao M., Zeng L.T. (2018). Retest of the relationship between air pollution and economic growth based on semiparametric spatial model. Stat. Res..

[35] Zheng W.J., Ye A.Z. (2015). Urban-rural income gap, industrial structure upgrading and economic growth-based on semi-parametric spatial panel VAR model. Economist.

